# Subjective Effects of Inhaling Kuromoji Tea Aroma

**DOI:** 10.3390/molecules26030575

**Published:** 2021-01-22

**Authors:** Eri Matsubara, Takeshi Morikawa, Norihisa Kusumoto, Koh Hashida, Naoyuki Matsui, Tatsuro Ohira

**Affiliations:** Forestry and Forest Products Research Institute, 1 Matsunosato, Tsukuba, Ibaraki 305-8687, Japan; tmorik@ffpri.affrc.go.jp (T.M.); nkusumoto@ffpri.affrc.go.jp (N.K.); koh@ffpri.affrc.go.jp (K.H.); naomat@ffpri.affrc.go.jp (N.M.); otatsu@ffpri.affrc.go.jp (T.O.)

**Keywords:** kuromoji, leaves, branches, Japanese herbal tea, subjective effects

## Abstract

Teas and various herbal teas are well-known beverages and are commonly consumed around the world. In this study, we focused on kuromoji tea. Kuromoji is a deciduous shrub of the Lauraceae family, and the plucked leaves and branches have been drunk as a tea in production areas for a long time. However, no studies have investigated the subjective and physiological effects of kuromoji tea. In this study, the effects of kuromoji tea were examined on the basis of the measurements of heart rate variability and cerebral blood flow, core body temperature and subjective assessments. Moreover, the results of this study showed that a pleasant subjective feeling could be obtained by sniffing the aroma of kuromoji teas, especially tea leaves. It was also found that the aroma of kuromoji teas has the potential to stimulate saliva secretion and increase subjective and physiological excitements in the oral cavity. 1,8-Cineole, linalool, terpinen-4-ol, carvone and geraniol were determined in both kuromoji leaves and branches. In this study, the beneficial effects of kuromoji teas when drunk conventionally were investigated.

## 1. Introduction

Tea is a well-known beverage and is produced from the dried leaves and stems of tea plants (*Camellia sinensis*). The type of tea is classified as black, oolong, or white, depending on the manufacturing process. Moreover, beverages not produced from tea plants are also broadly known as teas; many of these are produced from dried leaves and stems, fruits and flowers of various plants.

The relationships between the compounds in tea and their potential health benefits have been reported. Green tea and its main compounds—catechins, theanine and caffeine—have been indicated to have potentially beneficial effects (e.g., anticancer, anti-obesity, antioxidant, antidiabetic, anti-stress and anti-anxiety) [1,2,3,4] and cognitive and mood effects [5,6] in previous studies.

Furthermore, tea contains a large number of volatile compounds. These volatile compounds are deeply related to the subjective evaluation with which we decide whether we want to drink a tea or not before tasting it. Regarding the volatile compounds of tea, several previous studies have reported the relationship between volatiles and physiological and/or emotional responses. The inhalation of volatiles from green tea has the potential to induce more positive emotions and alter the brain processes [7,8]. In addition, black tea inhibits the increase of the salivary stress marker induced by arithmetic mental stress tasks [9]. Furthermore, the inhalation of volatiles from jasmine tea was also indicated to activate the parasympathetic nerves and induce a positive emotion [10].

Kuromoji (*Lindera umbellata*) is a deciduous shrub of the Lauraceae family and grows naturally across Japan. Kuromoji is classified into two kinds and three variants, and each kind has been reported to have different characteristic volatile compounds [11]. In addition, kuromoji is used as a raw material for producing high-quality toothpicks. The leaves and branches of kuromoji have been dried and consumed as a tea in production areas for a long time, and it is considered a traditional Japanese herbal tea. Regarding the effects of the volatile compounds of kuromoji, previous reports with essential oil and herbal water have suggested that it has subjective and physiological benefits in humans [12,13,14]. It was speculated that similar effects could be seen with kuromoji teas. However, no studies have yet investigated the subjective and physiological effects of kuromoji tea. Therefore, we make a hypothesis that the relaxing effects of the volatile compounds of kuromoji teas and we compared in healthy volunteers with kuromoji teas and control teas by the method of a randomized, crossover trial.

In the present study, kuromoji teas were evaluated in a manner close to their usual drinking context. The participant sniffed the aroma of the experimental material and then inhaled the same material while seated. During the experiment, to check the relaxing effect of the aroma, the changes in the participants’ heart rate variability were measured, and a questionnaire survey to assess the participants’ perception of the aroma was conducted. Additionally, to consider the other function of kuromoji teas, cerebral blood flow and core body temperature were measured. Furthermore, the aroma of kuromoji tea was qualitatively examined using headspace–solid-phase microextraction (HS-SPME)/gas chromatography–mass spectrometry (GC–MS) in order to analyze the components that were close to the aroma that humans perceived in the nasal cavity.

## 2. Results and Discussion

### 2.1. Analysis of Subjective Assessments

**Figure 1:**
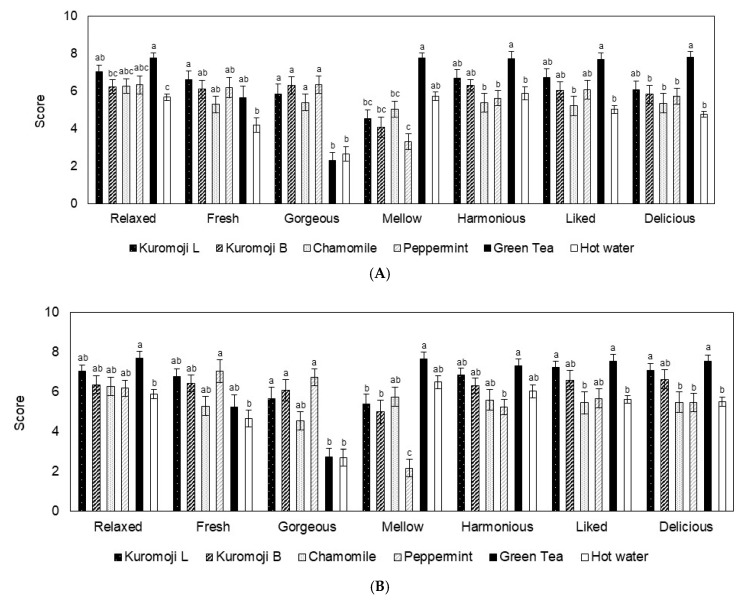

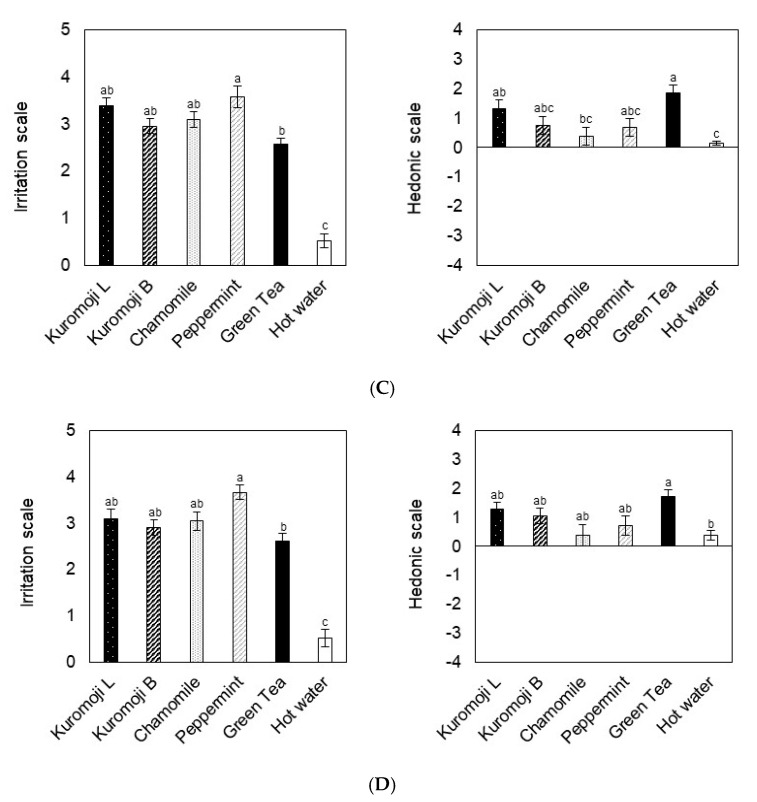
Subjective assessments of aroma for the experimental materials. Visual analog scale (VAS) scores in experiment 1 (**A**) and 2 (**B**) are shown. Irritation scores and hedonic scores in experiment 1 (**C**) and 2 (**D**) are also shown. Different letters represent statistically significant differences among experimental materials. Data are shown as means ± SEM.

The level of subjective responses when sniffing or inhaling each experimental material was determined using the visual analog scale (VAS), which is a seven-item questionnaire, an irritation scale and a hedonic scale. The results in experiments 1 and 2 are shown in Figure 1.

A difference in VAS scores within the experimental periods was shown only for the “mellow” and “delicious” items, with only peppermint and hot water. Between the test materials at different experimental periods, the VAS scores showed a significant difference in the kuromoji teas compared with other material teas. In experiment 1, for the “relaxed” item, the levels of kuromoji leaves were higher than those of hot water (*p* < 0.05), whereas the levels of kuromoji branches were lower than those of green tea (*p* < 0.05). For the “fresh” item, the levels of kuromoji leaves were higher than those of hot water (*p* < 0.05). For the “gorgeous” item, the levels of kuromoji leaves and branches were higher than those of green tea and hot water (*p* < 0.01). For the “mellow” item, the level of green tea was higher than that of kuromoji leaves and branches (*p* < 0.01). For the “delicious” item, the levels of green tea were higher than those of kuromoji branches (*p* < 0.05) (Figure 1A). On the other hand, in experiment 2, for the “gorgeous” item, the levels of kuromoji leaves and branches were higher than those of green tea and hot water (*p* < 0.01). For the “mellow” item, the levels of kuromoji leaves and branches were lower than those of green tea (*p* < 0.05), whereas the levels of kuromoji leaves and branches were higher than those of peppermint tea (*p* < 0.01, *p* < 0.05). For the “liked” item, the level of kuromoji leaves was higher than that of hot water (*p* < 0.05). For the “delicious” item, the level of kuromoji leaves was higher than that of hot water (*p* < 0.05) (Figure 1B).

The differences in both irritation levels and hedonic scales within the experimental periods are not shown. Changes in the irritation levels and hedonic scales between the test materials in different experimental periods were significantly different. The irritation levels (0–5) corresponded to “not at all”, “very slight”, “slight”, “distinct”, “strong” and “very strong” in the subjective evaluation. In experiments 1 and 2, the levels of kuromoji leaves and branches were higher than those of hot water (*p* < 0.01) (Figure 1C). The levels of the hedonic scales (−4 to 4) were “dislike extremely”, “dislike very much”, “dislike moderately”, “dislike slightly”, “neither like nor dislike”, “like slightly”, “like moderately”, “like very much” and “like extremely” in the subjective evaluation. In experiment 1, the level of kuromoji leaves was higher than that of hot water (*p* < 0.05) (Figure 1D).

In the present study, three questionnaires for the subjective assessment of the experiment materials were administered. The participants sniffed the aroma emitted from the materials in experiment 1 and inhaled the aroma through the mouth and nose in experiment 2. After each experiment, they answered the questionnaires administered. The VAS scores of the “relaxed”, “fresh”, and “gorgeous” items in the kuromoji leaves and “gorgeous” items in the kuromoji branches were significantly higher than those of green tea and/or hot water. Moreover, the scores of the “gorgeous”, “liked”, and “delicious” items in the kuromoji leaves and “gorgeous” items in the kuromoji branches were significantly higher than those of green tea and/or hot water. Furthermore, the VAS scores of the kuromoji leaves were similar to those of green tea.

**Figure 2:**
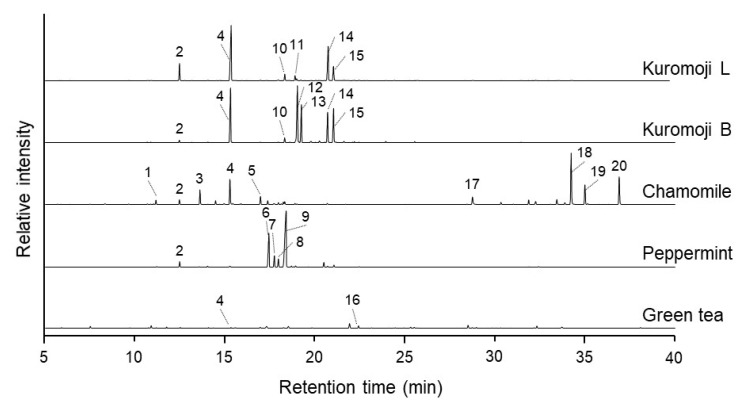
Headspace–solid-phase microextraction (HS-SPME)/gas chromatography–mass spectroscopy (GC–MS) chromatograms of the experimental materials used in this study. Numbers accompanying each peak indicate the peak number in Table 1.

These results showed that the aroma of kuromoji tea, especially kuromoji leaves, causes pleasant feelings such as comfort, freshness and deliciousness. Moreover, many Japanese people are accustomed to green tea, especially its aroma and taste. In this study, because many participants recognized the aroma and taste of green tea, the scores in the subjective evaluation of green tea were the highest in both experiments. In addition, the scores of the kuromoji leaves and green tea were very similar. Although kuromoji grows naturally across Japan and has been consumed as a tea in production areas for a long time, it was found that the participants were unfamiliar with it based on the interview survey. Previous studies indicated that the aroma of kuromoji branches tends to improve mood and reduce mental fatigue. They also reported that monoterpenes were the main compounds in the extracts of kuromoji branches, and linalool content was the largest among all monoterpenes [12,14]. In our studies, as shown in Figure 2 and Table 1, kuromoji teas contain 1,8-cineole, linalool, terpinen-4-ol, α-terpineol, *cis*- and *trans*-dihydrocarvones, carvone, geraniol. The subjective benefits of linalool and linalool-rich wine have also been reported in previous studies [15,16,17,18]. Furthermore, our results showed similar trends to previous reports, and we suggested that the aroma component of kuromoji teas, especially kuromoji leaves, promotes positive subjective effects.

### 2.2. Heart Rate Variability Analysis

The relative values of the normalized low frequency (LF) and high frequency (HF) (LF norm and HF norm, respectively) were calculated using the values of the experiment (for 10 s before the experiment). In experiment 2, the period of inhaling the aroma for each participant was different, so we used the data of the first 20 s to calculate the LF norm and HF norm. The average of these values for each experiment and the *p* values are shown in Table 2. The changes in both parameters were not significant for each of the experiment materials in each experiment. Meanwhile, between the experiments, the values of the LF norm were significantly different for the kuromoji leaves, peppermint tea and green tea (*p* < 0.05). Moreover, the values in the other tea conditions increased between the experiments, but no statistical significance was observed. The values in the HF norm decreased with no statistical significance, but those in hot water increased.

Sympathetic nervous activities significantly increased during experiment 2 after inhaling the aromas of kuromoji leaves, peppermint tea and green tea. These results showed that the sympathetic nervous activities were higher when inhaling the aroma through the mouth and nose than when simply sniffing the aroma of tea. The increase of such activities is known to be an index indicating subjective and physiological tension and excitement. Therefore, it can be indicated that the subjective and physiological excitement occurred when inhaling the aroma of these teas.

The aroma of foods is known to be perceived through two different routes: the orthonasal and retronasal routes [20,21]. In the orthonasal route, we sniff an aroma before eating, and this enters our nose through the nostrils, whereas in the retronasal route, we masticate and swallow the food when eating. Moreover, it has been reported that the reaction sites of the brain are different for the aromas from the two routes [22]. The retronasal olfaction refers to the perception of the aromas that escape from the back of the throat to the nasal cavity, which occurs not only when chewing food but also when drinking a beverage. The various influencing factors, such as the change in the composition of aromas due to the temperature in the oral cavity or the water-soluble nonvolatile component, have been suggested influencing the participant’s perception and subjective and physiological state. Previous studies have reported that sympathetic nerve activity is enhanced by tastants spread in the oral cavity [23,24]. In this study, the composition of the aroma components in the oral cavity was not analyzed. In the future, we would like to investigate the composition of aroma components in the oral cavity to reveal the difference in its influence on sympathetic nervous activity.

### 2.3. Analysis of Oxygenated Hemoglobin Changes

The relative oxygenated hemoglobin (oxyHb) values were calculated using the values of the experiment (for 30 s before the experiment). The average of these values in each experiment and the *p* values are shown in Table 3. The values in both sensor positions were not statistically significant for the experiment materials and between the experiments.

When neurons become active, the local blood flow to the relevant brain regions increases, and oxygenated blood displaces deoxygenated blood. The measurement of oxyHb values is most useful because the changes in oxyHb are the most sensitive indicators of the changes in regional cerebral blood flow among the three near-infrared spectroscopy (NIRS) parameters [25,26]. In this study, we focused on the blood flow changes measured in the temple because these changes have been suggested as being an indicator of salivary gland activity, particularly parotid activity [27,28,29]. The difference in the oxyHb values was not statistically significant between the tea conditions in both experiments 1 and 2, and no statistically significant difference was observed between the experiments either. However, the change in the oxyHb value when sniffing the aroma of kuromoji leaves was the largest on both left and right sides. Previous reports indicated that the aroma of kuromoji essential oil could promote salivary secretion [13]. On the basis of previous results and our results, it was inferred that the aroma of kuromoji materials has the potential to stimulate salivary gland activity and saliva secretion. However, the temples are part of the brain; in addition to the changes in blood flow associated with salivary gland activity, the nervous and muscle activities in the vicinity have the capacity to cause changes in blood flow. In this study, the volume of saliva in each participant after sniffing the aroma was not analyzed. We will attempt to confirm the effects of the aroma of kuromoji leaves on salivary gland activity in a future study.

### 2.4. Core Body Temperature Analysis

The relative values of core body temperature were calculated using the values of the experiment (for 10 s before the experiment). The average of these values for each experiment and the *p* values are shown in Table 4. The changes in all parameters were not significant between the experimental materials in each experiment.

Although it has been reported that the eardrum temperature increased when sniffing the aroma of whiskey and eating a hot meal [30,31], in this study, in experiment 2, the temperature was increased because of the hot beverage, but no significant difference was observed between the experimental materials.

### 2.5. Correlation between Subjective Assessments and Physiological Parameters

**Figure 3:**
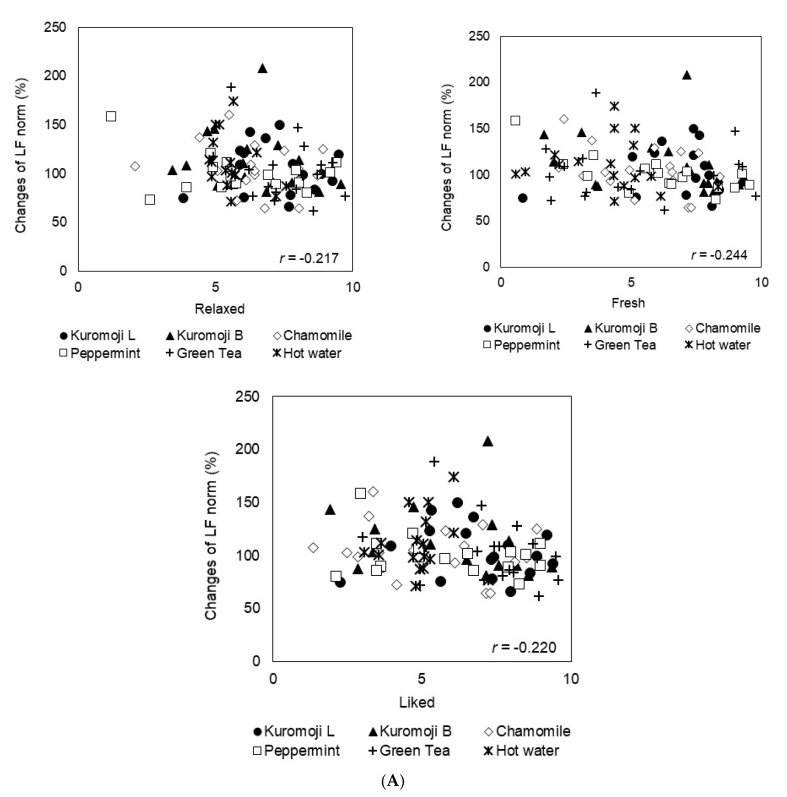

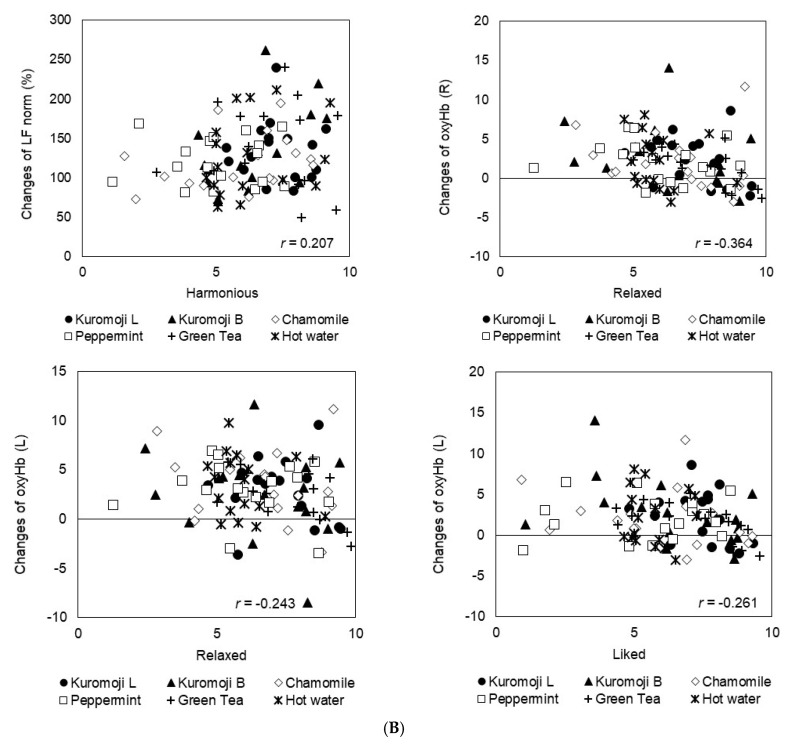
Relationships between subjective feelings and physiological responses. Plots represent the relationships between the values of LF norm or oxyHb and the subjective assessments in experiment 1 (**A**) and 2 (**B**).

A significant correlation in all teas is shown in Figure 3. In experiment 1, a negative correlation was observed between the relative values of the LF norm and the subjective assessments “relaxed” (*p* < 0.05), “fresh” (*p* < 0.05) and “liked” (*p* < 0.05) (Figure 3A). In experiment 2, a positive correlation was observed between the relative values of the LF norm and the subjective assessment “harmonious” (*p* < 0.05), and a negative correlation between the relative oxyHb values on the left and right sides and the subjective assessments “relaxed” (*p* < 0.01, 0.05) and “liked” (*p* < 0.01) (Figure 3B). Furthermore, no significant correlation was observed between the other parameters.

Regarding experiment 1, a significant correlation between the decrease of sympathetic nervous activity and increase of relaxation, freshness and the favoring of a specific tea in the subjective assessments was shown. Our results indicated that the aroma has the potential to induce physiological relaxation before drinking the tea. Many previous reports have indicated that sniffing a pleasant aroma could reduce sympathetic nervous activity and increase physiological relaxation [32,33,34,35].

Regarding experiment 2, a significant correlation between the increase of sympathetic nervous activity and the increase of harmonious feeling in the subjective assessments was shown. In addition, a significant correlation between the decrease of blood flow in the temples and increase of relaxation and the favoring of a specific tea in the subjective assessments was shown. In comparison with the results of experiment 1, a different correlation was observed when inhaling the aroma contained in the mouth. With regard to cerebral blood flow changes, a decrease in the oxyHb values was shown, despite the subjective feelings of favoring a certain tea. The brain is known to have different functions depending on the specific site; assuming that the amount of blood flowing in the brain is almost constant, in this study, the prefrontal cortex was transiently activated in order to judge the aroma and taste of tea contained in the mouth, and it could be suggested that, as a result, the blood in the temples was affected. Because we used only one sensor for each of the left and right sides, it was unclear if such a phenomenon occurred and if it caused a relative decrease in the blood flow rate in the temple.

In this study, we examined the correlations using all of the experimental materials, but in a future study, we would like to clarify the subjective and physiological effects of kuromoji teas.

This study has some limitations. First, the sample size used was small and limited to male university students. Since this experiment is a pilot study for a large-scale survey in the future, we conducted a model experiment for young men with relatively high uniformity. This limits the generalizability and applicability of the findings to the general population. Second, because we wanted to verify the effects in a natural way, similar to that when usually drinking, the method of inhalation was not limited, and the timing of breathing and swallowing for each tea was different for each participant. Therefore, it was possible to perform the analysis only for a short time. However, it is necessary to verify the influence of the kuromoji tea aroma components by the retronasal route, and we would like to improve the experimental system in the future.

### 2.6. Constituent Analysis of Experiment Materials

The volatile components in the tea samples were analyzed using the HS-SPME/GC–MS method. The results of the GC–MS analysis (total ion current chromatograms) are shown in Figure 2, and the identified compounds are listed in Table 1. Linalool, carvone, and geraniol, which were reported to be contained in kuromoji leaves [36], were identified in both kuromoji leaves and branches. In addition to these compounds, 1,8-cineole was also detected in kuromoji leaves, whereas *cis*- and *trans*-dihydrocarvones were detected in kuromoji branches. Similar to previous reports, it was confirmed that the flavor components in chamomile tea are α-bisabolol oxide B, α-bisabolone oxide A, and α-bisabolol oxide A [37], and those in peppermint tea are l-menthone and menthol [38]. In green tea, linalool and indole, which are the flavor constituents in manufactured green tea [39], were detected, but the amount detected was small in our method. We found that several specific compounds, terpinen-4-ol, α-terpineol, *cis*- and *trans*-dihydrocarvones, carvone and geraniol, were contained only in kuromoji leaves and branches. Previous reports have indicated that the essential oils containing terpinen-4-ol, α-terpineol and geraniol improve mood by short time inhalation [40,41,42,43], and these compounds have sedative activity on the central nervous system [44,45,46]. Therefore, these compounds in kuromoji teas were considered to have the potential for subjective and physiological activities, likewise linalool above.

## 3. Materials and Methods

### 3.1. Participants and Experimental Procedure

The experimental procedure of this study was approved by the Forestry and Forest Products Research Institute, and the study was performed in accordance with the Declaration of Helsinki. Twenty-one participants (all males, aged 21.3 ± 1.3 years (mean ± SD); age range: 19–24 years), all of whom were university students, were recruited for this research. They mainly preferred to drink green tea, oolong tea or barley tea. None of the participants had any physical or mental health conditions, and none were using prescription drugs or were smokers at the time of the study. Wearing strong fragrances and eating food with a strong smell were also forbidden the day before and on the day of the experiment.

The purpose and schedule of the experiments were explained to the participants. Written informed consent was obtained from all participants prior to the initiation of the study. Prior to the experiment, it was verified that the participants had a normal olfactory sense by the panel selection test (five standard aromas for selecting panel members, Daiichi Yakuhin Sangyo, Co., Ltd., Tokyo, Japan).

**Figure 4:**
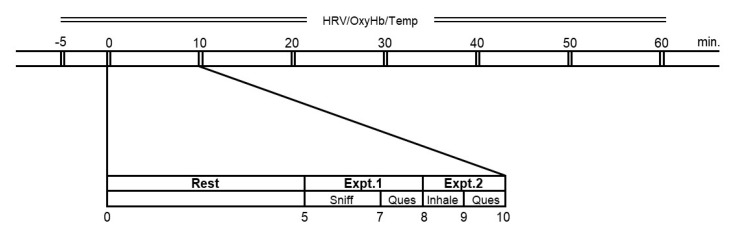
Experimental procedure. Heart rate variability, oxygenated hemoglobin and core body temperature were continually measured before, during and after the experiment. Questionnaires for subjective assessments, the irritation scale, the hedonic scale and the VAS were administered immediately after sniffing or inhaling the aroma of the experimental materials. Abbreviations are as follows: HRV, heart rate variability; OxyHb, oxygenated hemoglobin; Temp, core body temperature; Sniff, sniffing; Inhale, inhaling; Ques, questionnaires; Expt.1, experiment 1; Expt.2, experiment 2.

An experimental room in our research institute was used (an artificial climate chamber with the following interior dimension: a width of 3000 mm, depth of 4000 mm and height of 2500 mm). The experimental procedure is shown in Figure 4. After a 5 min rest, in experiment 1, the participants picked up a paper cup with the experimental material and sniffed the aroma of the experimental material. In experiment 2, they transferred the material from the paper cup to a glass cup, put the material in their mouth and inhaled the aroma. All participants performed both experiments six times with randomization of the order of experimental materials.

We used commercially available teas, kuromoji leaves and branches as experimental materials. To measure the subjective and physiological effects of the teas, immediately before each experiment, we put a teabag in a paper cup, poured 150 mL of 90 °C hot water and allowed the tea to steep for 5 min. The weight of each material in a tea bag is shown in Table 5. Chamomile tea, peppermint tea, green tea (Ban-cha) and hot water only were used as control materials. We purchased all tea from a Japanese tea shop (Yamaneen, Tokyo, Japan). Regarding the container with the experiment material, we used a paper cup with a lid in experiment 1 and a glass cup in experiment 2.

### 3.2. Subjective and Physiological Measurements

#### 3.2.1. Subjective Assessments

We used three questionnaires for the subjective evaluation of the aroma of the experiment materials: the VAS, irritation scale and hedonic scale. The VAS is a seven-item questionnaire designed to differentiate the subjective responses to the experiment materials, including “relaxed–irritated”, “fresh–stale”, “gorgeous–primitive”, “mellow–exciting”, “harmonious–disharmonious”, “liked–disliked” and “delicious–unappetizing.” Irritation to aroma was evaluated using a six-point Likert scale ranging from “none” to “excessively strong.” Hedonic responses were rated using a nine-point Likert scale ranging from “extremely pleasant” to “extremely unpleasant” [47]. Moreover, the questionnaires were administered immediately after sniffing or inhaling each experimental material in experiment 1 or experiment 2.

#### 3.2.2. Heart Rate Variability Analysis

We used a compact biological sensor (WHS-1, Union Tool Co., Tokyo, Japan) to measure the interval between heartbeats. The sensor was fixed to the chest of the participant and sampled from pre-work to the end of the experiment. The heart rate variability was analyzed using the RRI analyzer (Union Tool Co., Tokyo, Japan), and the autonomic nervous system responses were calculated using frequency analysis. For frequency analysis, both LF (0.04–0.15 Hz) and HF (0.15–0.4 Hz) components were analyzed, and the values of the LF norm and HF norm, which represent the value of each power component in proportion to the total power minus the very-low-frequency (VLF) component, were calculated. It is known that to minimize the influences of changes in the VLF component on total power, these values must emphasize the changes in the sympathetic and parasympathetic nerve activities.

#### 3.2.3. Oxygenated-Hemoglobin Changes Recording

NIRS is one of the neuroimaging techniques that operates under the principle that near-infrared light is absorbed by oxyHb and deoxygenated hemoglobin. The changes in oxyHb values were used as a parameter of neuronal activity against the sniffing or inhaling of the aroma of the experimental materials. We measured oxyHb values using a near-infrared oxygenation monitor (NIRO-200, Hamamatsu Photonics, Shizuoka, Japan). The sensors of NIRO-200 were composed of an illuminator and a detector, and before starting the experiment, we set the sensors on both temple parts of the participant. The changes in oxyHb values were recorded every 1 s during the experiment.

#### 3.2.4. Core Body Temperature Analysis

We used an earphone-type infrared thermometer (CE-Thermo, NIPRO, Osaka, Japan) to measure the core body temperature in the tympanic membrane. The sensor was fixed to the ear of the participant and sampled from pre-work to the end of the experiment. The continuous data were collected in a logger and then analyzed afterward.

### 3.3. HS-SPME/GC–MS Analysis

Volatile compounds emitted from each experimental material were analyzed using the SPME procedure. The 90 °C hot water extract of each material was filtrated and put into a 7 mL pierce vial. One milliliter of each sample was placed using a magnetic stirrer and tightly screw-capped with Mininert^®^ valves. Each vial was equilibrated in a 50 °C aluminum block for 5 min before extraction. Moreover, an SPME fiber (50/30 μm divinylbenzene/carboxen/polydimethylsiloxane (Supelco Co., PA, USA)) was exposed into the headspace of the vial and extracted for 20 min at 50 °C under stirring conditions. The SPME fibers were conditioned prior to use in a GC injector at 250 °C for 30 min.

The SPME fiber was immediately injected into the GC–MS system (GCMS-QP2010 Ultra; Shimadzu Co., Ltd., Kyoto, Japan) equipped with a DB-5 ms capillary column (30 m × 0.25 mm i.d., 0.25 μm film thickness; Agilent Technologies Ltd., CA, USA). The temperature program was set as follows: 40 °C for 3 min, which then increased to 200 °C at a rate of 4 °C/min for 0 min, and then increased to 280 °C at a rate of 16 °C/min for 2 min. Helium was used as the carrier gas at a flow rate of 2 mL/min with a split ratio of 1:20, the injector temperature was 240 °C, and the detector temperature was 200 °C. Mass spectra were recorded over a 40–300 amu range at 3.3 scans per second with ionization energy of 70 eV. Each component was identified by comparing its mass spectra with a mass spectral library (NIST14 and FFNSC3), and retention indices were calculated from a series of *n*-alkanes (C_9_–C_20_) and compared with those in the literature [19].

### 3.4. Statistical Analysis

All values were expressed as means ± SEM. Mann–Whitney *U* test and Kruskal–Wallis tests were performed to compare within and between-material differences in the subjective assessment of the VAS questionnaire and the irritation and hedonic scales, respectively. A one-way ANOVA with post hoc comparisons using the Bonferroni test and Student’s t-test was conducted to compare the differences in physiological parameters between experimental materials and experiments. Multivariable analysis with spearman’s correlation was performed to consider the correlation between the subjective and physiological parameters. Moreover, the results were considered statistically significant when *p* was <0.01 or <0.05. All statistical analyses were performed using SPSS 26.0 J for Windows (SPSS Japan, Tokyo, Japan).

## 4. Conclusions

In the present study, the effects of the kuromoji leaves and branches were investigated using a model experiment. The participants were encouraged to sniff the aroma of the experimental materials and rate their perceptions of the aroma. The changes in the participants’ heart rate variability, cerebral blood flow and core body temperature were measured. The results showed that a pleasant subjective feeling could be obtained by sniffing the aroma of the kuromoji tea, especially kuromoji leaves. In addition, it was found that the aroma of the kuromoji tea has the potential to stimulate the activity of the salivary gland and increase subjective and physiological excitement. On the basis of the results, it was possible to the functionality of kuromoji teas.

## Figures and Tables

**Table 1:** Major volatiles identified in the experimental materials by SPME-GC–MS analysis.

Peak	Compound	RI_ref_^a^	RI ^b^	SI ^c^
1	Yomogi alchool	999	994	95
2	1,8-Cineole	1026	1027	92
3	Artemisia ketone	1056	1055	99
4	Linalool	1095	1098	96
5	Camphor	1141	1140	96
6	l-Menthone	1148	1150	97
7	Isomenthone	1158	1160	98
8	Neomenthol	1161	1166	95
9	Menthol	1167	1173	97
10	Terpinen-4-ol	1174	1175	91
11	α-Terpineol	1186	1190	95
12	*cis*-Dihydrocarvone	1191	1192	97
13	*trans*-Dihydrocarvone	1200	1198	97
14	Carvone	1239	1239	97
15	Geraniol	1249	1247	94
16	Indole	1290	1285	81
17	1-Dodecanol	1469	1471	95
18	α-Bisabolol oxide B	1656	1649	94
19	α-Bisabolone oxide A	1684	1675	94
20	α-Bisabolol oxide A	1748	1741	87

^a^ Retention indices (RI) mentioned in [19]. ^b^ Retention indices calculated in this study. ^c^ Similarity indices (SI) compared with mass spectral libraries (NIST14 and FFNSC3).

**Table 2:** Relative values of normalized low and high frequencies.

Items	Experimental Materials	Expt.1	Expt.2	*p* Value
LF norm	Kuromoji L	104.1 ± 5.7	132.7 ± 8.5	0.011
	Kuromoji B	110.3 ± 7.5	125.1 ± 12.1	0.315
	Chamomile	106.6 ± 6.0	126.8 ± 10.0	0.101
	Peppermint	99.3 ± 4.4	120.6 ± 6.8	0.016
	Green Tea	104.0 ± 7.2	142.9 ± 12.5	0.013
	Hot water	110.4 ± 6.3	125.8 ± 11.2	0.250
HF norm	Kuromoji L	93.4 ± 5.2	85.3 ± 7.2	0.378
	Kuromoji B	103.4 ± 9.5	92.3 ± 7.2	0.362
	Chamomile	93.8 ± 5.1	85.8 ± 13.1	0.583
	Peppermint	101.8 ± 6.9	92.0 ± 10.3	0.440
	Green Tea	92.1 ± 8.1	82.8 ± 10.0	0.486
	Hot water	97.8 ± 5.4	106.1 ± 10.5	0.499

Data are shown as means ± SEM. Abbreviations are as follows: LF norm, normalized low frequency; HF norm, normalized high frequency; Expt.1, experiment 1; Expt.2, experiment 2.

**Table 3:** Relative values in oxygenated hemoglobin.

Items	Experimental Materials	Expt.1	Expt.2	*p* Value
OxyHb at right side	Kuromoji L	3.7 ± 0.8	2.5 ± 0.6	0.275
	Kuromoji B	2.2 ± 0.5	2.7 ± 0.8	0.648
	Chamomile	2.4 ± 0.5	2.1 ± 0.7	0.702
	Peppermint	2.3 ± 0.4	2.0 ± 0.5	0.694
	Green Tea	2.7 ± 0.5	1.8 ± 0.5	0.254
	Hot water	3.0 ± 0.6	2.6 ± 0.7	0.653
OxyHb at left side	Kuromoji L	4.3 ± 0.7	3.4 ± 0.7	0.396
	Kuromoji B	2.2 ± 0.6	3.0 ± 0.9	0.535
	Chamomile	3.4 ± 0.6	3.6 ± 0.8	0.782
	Peppermint	3.1 ± 0.5	3.1 ± 0.6	0.995
	Green Tea	3.3 ± 0.4	2.8 ± 0.6	0.496
	Hot water	3.9 ± 0.6	4.0 ± 0.7	0.920

Data are shown as means ± SEM. Abbreviations are as follows: OxyHb, oxygenated hemoglobin; Expt.1, experiment 1; Expt.2, experiment 2.

**Table 4:** Relative values for core body temperature.

Items	Experimental Materials	Expt.1	Expt.2	*p* Value
Core body temperature	Kuromoji L	100.0 ± 0.05	100.0 ± 0.02	0.646
	Kuromoji B	99.9 ± 0.05	100.0 ± 0.01	0.291
	Chamomile	100.0 ± 0.04	99.9 ± 0.05	0.317
	Peppermint	100.0 ± 0.05	100.0 ± 0.02	0.935
	Green Tea	99.9 ± 0.05	100.0 ± 0.01	0.509
	Hot water	99.9 ± 0.04	100.0 ± 0.02	0.078

Data are shown as means ± SEM. Abbreviations are as follows: Expt.1, experiment 1; Expt.2, experiment 2.

**Table 5:** Experimental materials.

Experimental Materials	Sites	Weight/Bag
Kuromoji (*Lindera umbellata*) tea	Leaves	2 g
Kuromoji (*Lindera umbellata*) tea	Branches	5 g
Chamomile (*Matricaria chamomilla*) tea	Flowers	2 g
Peppermint (*Mentha x piperita*) tea	Leaves	2 g
Green tea	Leaves	2 g
Hot water	―	―

## Data Availability

The data presented in this study are available on request from the corresponding author. The data are not publicly available due to restrictions, e.g., privacy or ethical.

## Sample Availability

Samples of the compounds are not available from the authors.
